# LncRNA PCBP1-AS1-mediated AR/AR-V7 deubiquitination enhances prostate cancer enzalutamide resistance

**DOI:** 10.1038/s41419-021-04144-2

**Published:** 2021-09-20

**Authors:** Boya Zhang, Mingpeng Zhang, Chunyi Shen, Guancong Liu, Fan Zhang, Jingyu Hou, Weitao Yao

**Affiliations:** 1 Department of Bone and Soft Tissue Oncology, Affiliated Cancer Hospital of Zhengzhou University, Zhengzhou, P. R. China; 2 Department of Urology, Tianjin Medical University Second Hospital, Tianjin, P. R. China; 3 Biotherapy Center, The First Affiliated Hospital of Zhengzhou University, Zhengzhou, P. R. China

**Keywords:** Prostate cancer, Ubiquitylation, Long non-coding RNAs

## Abstract

The refractory of castration-resistant prostate cancer (CRPC) is mainly reflected in drug resistance. The current research on the resistance mechanism of CRPC is still in its infancy. In this study, we revealed for the first time the key role of LncRNA PCBP1-AS1 in CRPC drug resistance. Through detailed in vivo and in vitro studies, we found that PCBP1-AS1 may enhance the deubiquitination of AR/AR-V7 by stabilizing the USP22-AR/AR-V7 complex, thereby preventing AR/AR-V7 from being degraded through the ubiquitin–proteasome pathway. Targeting PCBP1-AS1 can significantly restore the drug sensitivity of enzalutamide-resistant tumors in vivo and in vitro. Our research further expands the function of LncRNA in castration-resistant prostate cancer, which may provide new potential for clinical diagnosis and targeted therapy.

## Introduction

As the most common malignant tumor in the urinary system, the treatment of prostate cancer has always been the focus of research [1]. At present, androgen-deprivation therapy (ADT) is one of the main treatments [2–4]. According to the tumor’s ADT response, prostate cancer can be divided into hormone-sensitive prostate cancer (HSPC) and castration-resistant prostate cancer (CRPC). Although most patients can benefit from ADT treatment, after 2–3 years of ADT treatment, most patients will relapse and progress to CRPC [5, 6]. This not only indicates the recurrence of the tumor but also represents the failure of ADT treatment. Due to continuous in-depth research, drugs target AR, such as enzalutamide, can continue to benefit patients [7, 8]. But with the wide expansion of new drugs, the subsequent drug resistance has become increasingly prominent [9].

The continuous abnormal activation of the AR signal pathway is an important factor in the progress of CRPC. At present, one of the main treatment methods for CRPC is to suppress tumors by targeting AR, such as enzalutamide. However, CRPC tumor cells through AR point mutations, AR amplification, changes of androgen biosynthesis, and other mechanisms make CRPC resistant to drugs such as enzalutamide [10]. In recent years, AR splice variants have gradually received attention, which has made researchers realize that the factors that affect the progress of CRPC include not only full-length AR but also AR splicing variants, such as AR-V7 [5, 10, 11]. Although enzalutamide can more comprehensively inhibit the AR signaling pathway in CRPC by targeting AR synthesis, nuclear transport, and other processes, when resistance is formed, with the upregulation of AR and AR-V7, this inhibitory effect will no longer exist [12, 13]. Enzalutamide-resistant CRPC is generally refractory, and the prognosis of patients is significantly worse. One of the mechanisms is the reduction of AR and AR-V7 degradation. With the progress of research, it has been found that a variety of factors are involved in the regulation of the AR signaling pathway in CRPC, including long noncoding RNA (LncRNA), which has been found to be active in malignant tumors in recent years.

LncRNA is larger than 200 nt in length, usually cannot be translated into protein, and plays an important regulatory role in a variety of biological processes. In recent years, the importance of LncRNA in prostate cancer has been repeatedly mentioned. PCA3 has become an important indicator in the diagnosis of prostate cancer [14]. Both HOTAIR and PCAT1 have been proved to play important roles in the development of CRPC [15, 16]. In this study, we found that LncRNA PCBP1-AS1 was significantly increased in CRPC. Targeting PCBP1-AS1 in vitro can significantly inhibit the proliferation and migration of CRPC cell lines. Inhibiting PCBP1-AS1 in vivo can significantly inhibit the growth of tumors. After digging into the mechanism, we found that PCBP1-AS1 can stabilize the USP22-AR/AR-V7 complex, enhance AR/AR-V7 deubiquitination, and prevent the protein from being degraded through the ubiquitin–proteasome pathway by binding at the NTD domain of AR/AR-V7. Subsequently, this regulatory mechanism of PCBP1-AS1 was also proved to be closely related to the enzalutamide resistance of CRPC in vivo and in vitro. Targeting PCBP1-AS1 can not only significantly restore the sensitivity of drug-resistant tumors to enzalutamide but also narrow the tumor volume and growth. Our research expanded the importance of LncRNA in CRPC ubiquitination-related processes may provide new potential for clinical diagnosis and targeted therapy.

## Results

### PCBP1-AS1 is significantly related to clinical characteristics in CRPC

**Fig. 1:**
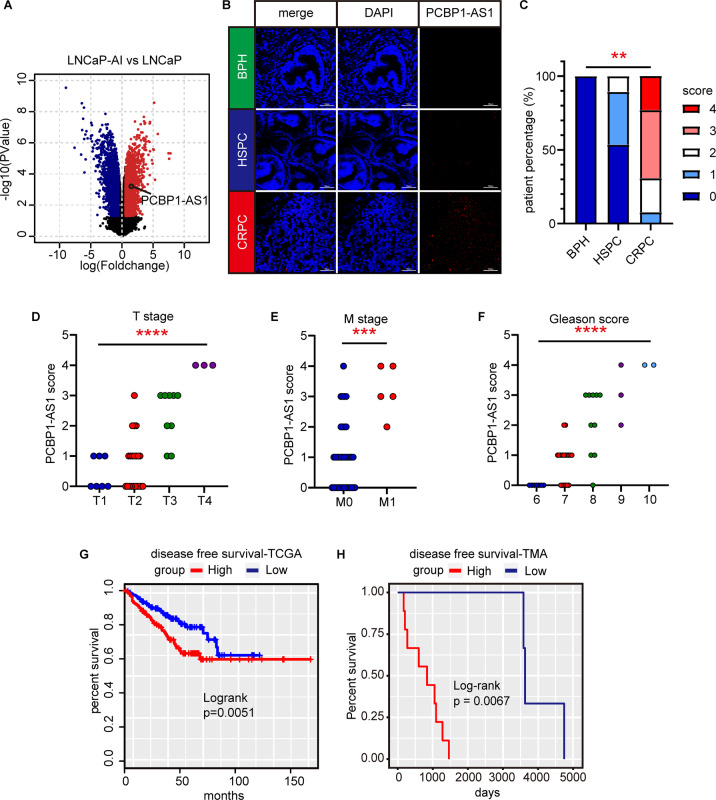
Clinical investigation of PCBP1-AS1 in prostate cancer. **A** Volcano plot of gene expression fold change and *P* value in GSE124291 (LNCaP-AI versus LNCaP). Red dots refer to genes upregulated in LNCaP-AI, blue dots refer to genes upregulated in LNCaP. **B** RNA-Scope results using TMA. Red dots in the “PCBP1-AS1” column refer to the expression of PCBP1-AS1 in indicated patient tissue. **C** Summary of the score in RNA-scope. The scoring is carried out in accordance with ACD’s corresponding specification standards. **D**–**F** The comparison results of T stage, M stage, and Gleason score of patients in TMA. ANOVA is used to calculate *P* value. **G**, **H** Kaplan–Meier survival plot of patients in TCGA database and TMA. Groups are divided by the median expression or RNA-scope score of PCBP1-AS1. Significance: *P<0.05, **P<0.01, ***P<0.001, ****P<0.0001.

In order to make a more comprehensive landscape of the noncoding RNA expression that occurred during the development of CRPC, we used the data from GSE124291 to analyze. This dataset established castration-resistant LNCaP-AI cell line by culturing LNCaP cells in a castration environment, so it can be regarded as a simulation of the process of HSPC (LNCaP) to CRPC (LNCaP-AI) in an in vitro environment. After setting the threshold (*P* value <0.05, |Foldchange | >2) and screening the results, we found that PCBP1-AS1 was significantly upregulated in CRPC (Fig. 1A). To confirm the result from the microarray, we used RNA-scope to detect PCBP1-AS1 expression in a patient tissue microarray (TMA, Benign prostatic hyperplasia (BPH) = 4, HSPC = 28, CRPC = 12, Table 1). BPH samples were used to show the expression of PCBP1-AS1 in benign lesions, to present a better landscape of PCBP1-AS1. We found that PCBP1-AS1 expression is low in BPH and relatively high in HSPC and CRPC. After quantifying the results, we found that the expression of PCBP1-AS1 in CRPC is significantly higher from HSPC. This suggests that PCBP1-AS1 may play an important role in CRPC (Fig. 1B, C). Subsequently, through the integration of clinical information, we found that in prostate cancer patients, the expression of PCBP1-AS1 was significantly positively correlated with the T stage (Fig. 1D), M stage (Fig. 1E), and Gleason score (Fig. 1F). This suggests that high PCBP1-AS1 expression may indicate a poor prognosis for patients. The subsequent Kaplan–Meier survival curve from TCGA and our own data also proved this point (Fig. 1G, H). The above data suggest that PCBP1-AS1 is closely related to the clinical features of prostate cancer and may play an important role in CRPC.

**Table 1:** PCBP1-AS1 clinical significance in TMA.

Characteristic	number	HSPC/CRPC	PCBP1-AS1 score (average)	*P* value
Disease type				
BPH	4		0.25	
HSPC	28		0.61	
CRPC	12		2.92	
Gleason				
6	7	7/0	0	0.02102*
7	18	15/3	0.78	
8	10	5/5	2.1	
9	3	1/2	3	
10	2	0/2	4	
T stage				
T1	7	7/0	0.43	0.006701*
T2	21	19/2	0.76	
T3	9	2/7	2.33	
T4	3	0/3	4	
N stage				
N0	29	24/5	0.83	0.2224**
N1	11	4/7	2.55	
M stage				
M0	35	27/8	1.03	0.01172**
M1	5	1/4	3.2	

^*^Pearson’s Chi-squared test.

^**^Pearson’s Chi-squared test with Yates’ continuity correction.

Significance threshold: 0.05, characteristics with *P* value < 0.05 are considered to be significantly related with PCBP1-AS1 expression and relavant *P* values are displayed in bold.

### Target PCBP1-AS1 repress cell proliferation, migration, and tumor growth

**Fig. 2:**
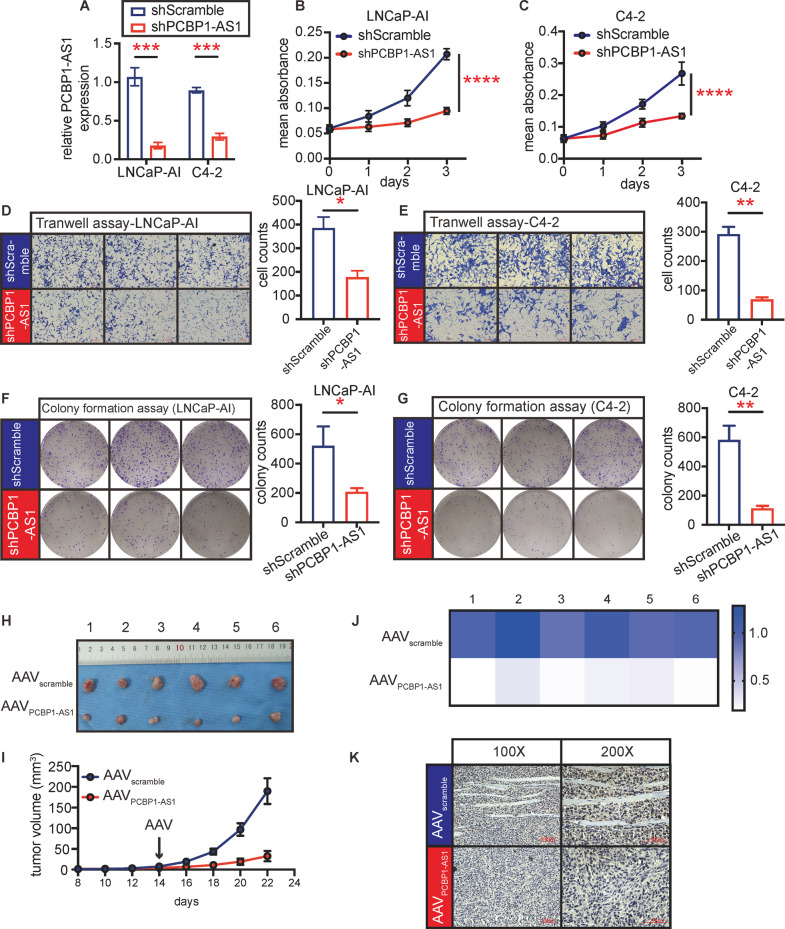
Targeting PCBP1-AS1 significantly affects tumor biological functions in vivo and in vitro. **A** Knockdown efficiency of PCBP1-AS1 in LNCaP-AI and C4-2 cell lines. **B**, **C** Cell viability detection by MTT assay. The *P* value at day 3 was calculated. **D**, **E** Cell migration detection by Transwell assay. The left panel performed indicated groups’ graph, right panel is the summary of cell counts. **F**, **G** Cell proliferation detection by colony formation assay. The left panel performed indicated groups’ graph, right panel is the summary of colony counts. **H** Graph of transplant tumors in in vivo experiment. **I** Tumor growth rate of in vivo experiment. Tumor volume was calculated at the scale of mm^3^. **J** PCBP1-AS1 expression in indicated groups. Expression was detected by qPCR. Panels **A**–**G** repeated three times, panels **H**–**K** repeated two times, with six biological replicates each time. Significance: *P<0.05, **P<0.01, ***P<0.001, ****P<0.0001.

Simulating the development of CRPC as realistically as possible helps us understand the changes more accurately. Therefore, we used C4-2 cells and castration-resistant LNCaP-AI cells evolved from LNCaP under long-term androgen deprivation (details in “Materials and methods”) for the next research. We used lentivirus targeting PCBP1-AS1 to knock down PCBP1-AS1 in LNCAP-AI and C4-2 cells (Fig. 2A). Subsequent cell function experiments showed that compared with the control group (shScramble), PCBP1-AS1 depletion (shPCBP1-AS1) can significantly inhibit the proliferation and migration ability of tumor cells (Fig. 2B–G). This suggests that PCBP1-AS1 plays a significant role in CRPC cell lines in in vitro conditions.

In order to further stimulate the situation in vivo, we performed subcutaneous tumor transplantation on nude mice with LNCaP-AI and treated with adeno-associated virus-containing scramble sequence (AAV_scramble_) or shPCBP1-AS1 sequence (AAV_PCBP1-AS1_). The results after 3 weeks of tumor growth showed that compared with AAV_scramble_, the tumor growth rate and tumor volume in the AAV_PCBP1-AS1_ group were significantly decreased (Fig. 2H, I). PCBP1-AS1 knockdown efficiency was confirmed by qPCR (Fig. 2J), and through IHC, we can see that the Ki-67 expression after PCBP1-AS1 depletion was significantly decreased (Fig. 2K). However, we also found that the effect of PCBP1-AS1 depletion seems to be limited to delaying the growth of the tumor but does not reduce the size of tumor. In any case, our results show that targeting PCBP1-AS1 can inhibit cell proliferation, migration in vitro, and inhibit tumor growth in vivo.

### PCBP1-AS1 function through regulating AR ubiquitin–proteasome degradation

**Fig. 3:**
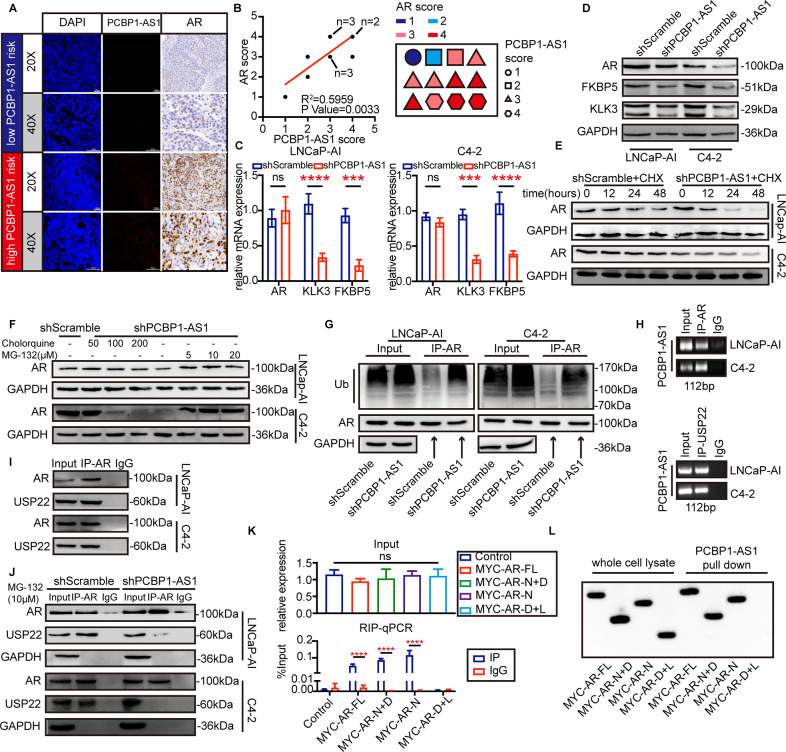
PCBP1-AS1 promotes USP22-AR stability by binding to AR NTD domain and enhances AR deubiquitination. **A** comparison of AR IHC and PCBP1-AS1 RNA-scope. All graphs present the same location of the same patients. **B** Left panel presents the Pearson’s correlation value *R*^2^ and *P* value of PCBP1-AS1 RNA-scope score and AR IHC score. Right panel presents the number, PCBP1-AS1 score, and AR score of each CRPC patient. The score was given by three experimental pathologists. **C** qPCR showed the expression of AR, KLK3, and FKBP5 RNA expression in LNCaP-AI and C4-2 cells before and after PCBP1-AS1 knockdown. **D** Western blot presents the expression of AR, KLK3, and FKBP5 protein expression in LNCaP-AI and C4-2 cells before and after PCBP1-AS1 knockdown. **E** AR protein degradation rate. CHX concentration: 100 µM. **F** AR protein expression after chloroquine or MG-132 addition. **G** AR ubiquitination-level detection after co-IP using AR antibody. GAPDH was used as input loading control. **H** RIP assay using AR antibody, followed by PCR to detect PCBP1-AS1 RNA expression. **I** Co-IP results using AR IP antibody and USP22 WB antibody. **J** Detection of AR-USP22 binding ability before and after PCBP1-AS1 knockdown. GAPDH was used as a loading control. **K** RIP-qPCR results present the binding results of PCBP1-AS1 with indicated plasmids. **L** RNA pull-down results using PCBP1-AS1 biotinylation probe and indicated AR plasmids. Panels **C**–**L** were repeated three times. Significance: *P<0.05, **P<0.01, ***P<0.001, ****P<0.0001.

We next obtained all the relevant genes in the “prostate cancer” signaling pathway (map05215) from the KEGG database and applied RPI-Seq algorithm to find proteins that may have a potential relationship with PCBP1-AS1 [17]. The results showed that PCBP1-AS1 has a high binding potential with AR protein (Table 2). Subsequently, we used the same series of TMA to perform immunohistochemistry (IHC) on the protein expression of AR in the same patient and compared the results with the previous PCBP1-AS1 RNA-scope (Fig. 3A). The RNA-scope score is significantly positively correlated with the IHC score of AR, which also verifies the results of RPI-Seq (Fig. 3B). In addition, after knocking down PCBP1-AS1, we observed that at the RNA level, AR did not show any change, but KLK3 and FKBP5 were significantly lower, while at the protein level, AR decreased significantly with PCBP1-AS1 depletion (Fig. 3C, D). This suggests a potential of AR post-transcriptional regulation by PCBP1-AS1.

**Table 2:** Top ten PCBP1-AS1-binding proteins*.

Protein	RF classifier	SVM classifier
AR	0.85	0.83
KRAS	0.85	0.66
SRD5A2	0.8	0.81
HRAS	0.8	0.6
MAP2K2	0.75	0.84
MAP2K1	0.75	0.8
NRAS	0.75	0.6
KLK3	0.7	0.77
HSP90	0.65	0.82
AKT	0.55	0.83

*RF* Random Forest, *SVM* support vector machine.

*Ranked by “RF classifier”.

Post-transcriptional regulation includes two parts: protein synthesis and protein degradation, so we use cycloheximide (CHX) to inhibit protein synthesis with different time gradients. As can be seen from Fig. 3E, AR expression decreased significantly at 24 h and 48 h after PCBP1-AS1 depletion. These results suggest that PCBP1-AS1 depletion significantly accelerates the degradation of AR protein after inhibiting protein synthesis. Subsequently, we used different concentrations of chloroquine or MG-132 to identify the degradation pathways regulated by PCBP1-AS1, we found that the degradation rate did not change after chloroquine treatment, but as the concentration of MG-132 increased within a certain range, the expression of AR gradually increased, suggesting that PCBP1-AS1 may regulate AR through the ubiquitin–proteasome pathway (Fig. 3F). To confirm our hypothesis, we evaluated AR ubiquitination level after being enriched by immunoprecipitation, and the results showed that after knocking down PCBP1-AS1, the ubiquitination level of AR protein increased significantly, which is consistent with our previous experiments (Fig. 3G). The above results suggest that PCBP1-AS1 may be involved in the ubiquitin–proteasome degradation process of AR.

### PCBP1-AS1 enhances USP14-AR complex stability by binding to the AR NTD domain

Previous experiments have confirmed that the expression of PCBP1-AS1 can inhibit the ubiquitination and degradation of AR, suggesting a potential relationship between PCBP1-AS1 and AR deubiquitination. Therefore, we hypothesized that PCBP1-AS1 can bind to members of the USP protein family of deubiquitinating enzymes. We selected USP14, USP22, and USP26 that have related studies for verification [18–20]. After RNA immunoprecipitation (RIP), we found that only USP22 can bind to PCBP1-AS1 (Fig. 3H). Subsequent immunoprecipitation also confirmed the association between AR and USP22 in vitro, which is consistent with the result of previous studies (Fig. 3I). At the same time, we found that after PCBP1-AS1 depletion in vitro, the binding ability of USP22 and AR decreased significantly (Fig. 3J).

The above results indicate that PCBP1-AS1 may be involved in stabilizing the USP22-AR complex. In order to further understand the combination of PCBP1-AS1 and AR, we constructed plasmids containing different AR domains: full-length AR (MYC-AR-FL), NTD, and DBD domains (MYC-AR-N + D), the plasmid contains only the NTD domain (MYC-AR-N) or both the DBD and LBD domains (MYC-AR-D + L). MYC tags were added to facilitate subsequent detection. We verified the binding region through RIP-qPCR and RNA pull-down experiments. The results suggest that PCBP1-AS1 binds to the NTD domain of AR, but not DBD or LBD domain (Fig. 3K, L).

### PCBP1-AS1 rescue restores tumor cell phenotype and enhances AR deubiquitination

**Fig. 4:**
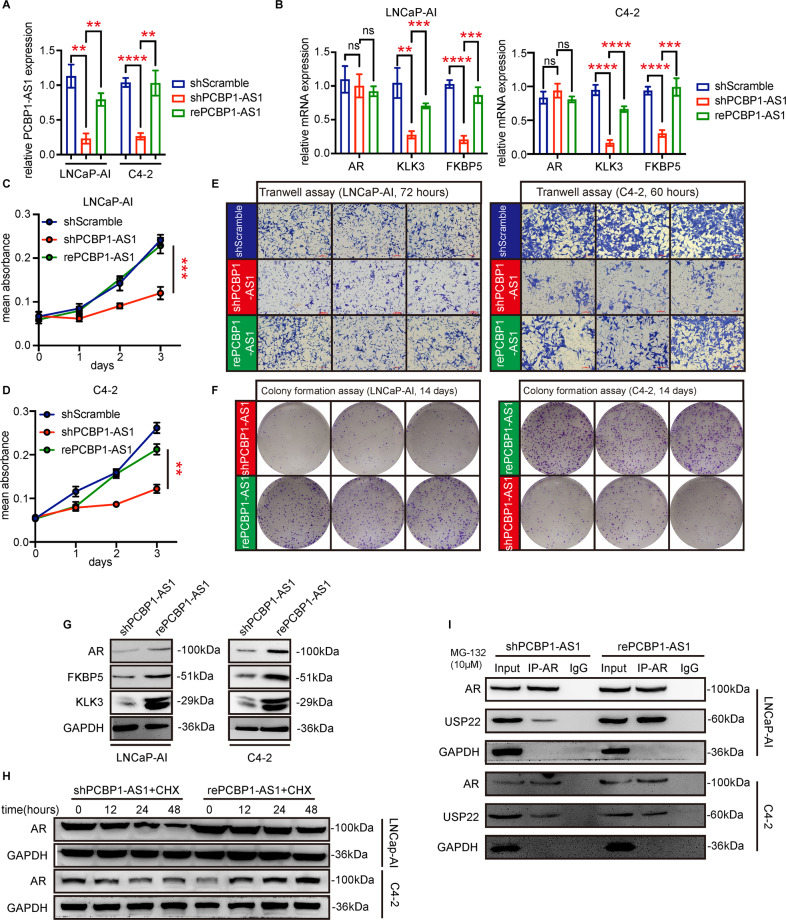
Rescue PCBP1-AS1 recover cell phenotypes and AR-USP22 complex stability. **A** PCBP1-AS1 rescue efficiency. GAPDH was used as a control. **B** AR, KLK3, FKBP5 RNA expression in indicated groups. **C**, **D** Cell viability detection using MTT in indicated groups. **E** Cell migration ability detected by Transwell assay in indicated groups. **F** Cell proliferation ability detected by colony formation assay in indicated groups. **G** AR, KLK3, FKBP5 protein expression before and after PCBP1-AS1 rescue. **H** AR degradation rate after CHX addition in indicated groups. **I** Co-IP was used to detect the binding ability between AR and USP22 in indicated groups. All experiments were repeated at least three times. Significance: *P<0.05, **P<0.01, ***P<0.001, ****P<0.0001.

Based on the above results, we performed PCBP1-AS1 rescue on the LNCaP-AI and C4-2 cells of shPCBP1-AS1 (Fig. 4A). After the successful overexpression of PCBP1-AS1, consistent with our hypothesis, the cell proliferation and migration ability were significantly restored (Fig. 4C–F), the mRNA expression of KLK3 and FKBP5 increased significantly, and the protein expression of AR and corresponding target genes was also alleviated (Fig. 4B, G). The subsequent detection of AR degradation rate also showed that PCBP1-AS1 rescue can effectively reduce the degradation of AR protein (Fig. 4H). The combination of USP22 and AR also increased significantly with the rescue of PCBP1-AS1 (Fig. 4I). These data indicate that PCBP1-AS1 can participate in stabilizing the USP22-AR complex to enhance AR deubiquitination and promote the progress of CRPC.

### Targeting PCBP1-AS1 represses AR-V7 expression and enhances enzalutamide response in CRPC

**Fig. 5:**
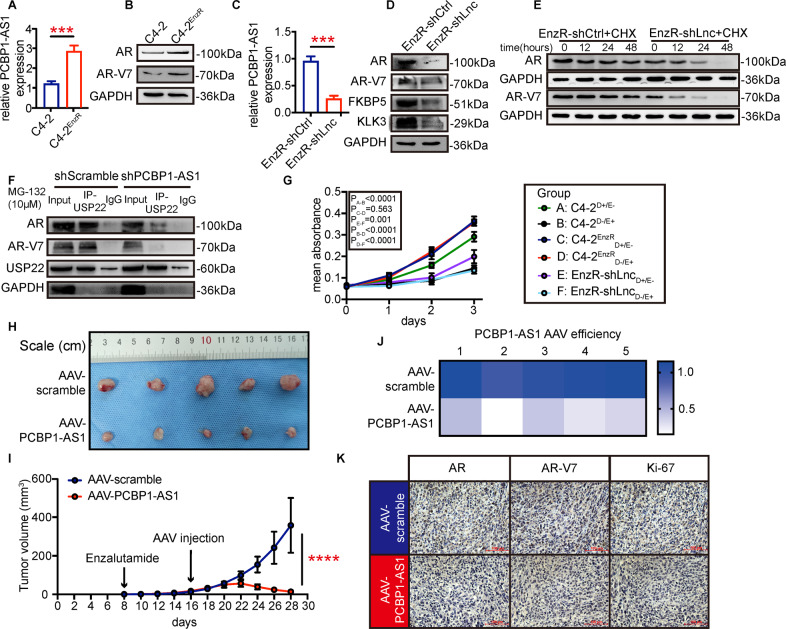
Targeting PCBP1-AS1 repress USP22-AR-V7 stability and CRPC enzalutamide resistance. **A** PCBP1-AS1 expression in C4-2 and C4-2-EnzR cells. **B** AR, AR-V7 expression in C4-2 and C4-2-EnzR cells. **C** PCBP1-AS1 knockdown efficiency in C4-2-EnzR cells. **D** AR, AR-V7, KLK3, FKBP5 expression before and after PCBP1-AS1 knockdown. **E** AR, AR-V7 degradation rate after CHX addition in indicated groups. **F** AR-USP22, AR-V7-USP22 binding ability detection using the co-IP assay. **G** Cell viability detected by MTT in indicated groups. **H** Enzalutamide-resistant tumors of the in vivo experiments. **I** Tumor growth rate of indicated groups. **J** PCBP1-AS1 expression detected by qPCR, present by heatmap. **K** AR, AR-V7, and Ki-67 expression in indicated groups, presented by IHC. Panels **A**–**G** were repeated three times, panels **H**–**K** were repeated two times, with five biological replicates each time. Significance: *P<0.05, **P<0.01, ***P<0.001, ****P<0.0001.

Based on previous research and our conclusions, we hypothesize that PCBP1-AS1 can regulate AR-V7, which also contains a complete NTD domain, through the same mechanism. In addition, the abnormal activation of the AR signaling pathway is closely related to the failure of enzalutamide treatment, so we induced enzalutamide-resistant C4-2 cells (C4-2^EnzR^) and set out to verify our hypothesis (details in “Materials and methods”). we found that compared with C4-2, the expression of PCBP1-AS1, AR, and AR-V7 in C4-2^EnzR^ cells was further significantly improved (Fig. 5A, B). After knocking down PCBP1-AS1 in C4-2^EnzR^ (EnzR-shLnc, EnzR-shCtrl is negative control), the expression of AR, AR-V7, KLK3, and FKBP5 all decreased significantly (Fig. 5C, D), and the degradation rate of AR and AR-V7 increased (Fig. 5E). The results of immunoprecipitation showed that PCBP1-AS1 depletion can significantly reduce the combination of AR-V7 and USP22 (Fig. 5F).

On the other hand, we divided C4-2 and C4-2^EnzR^ cells into six groups according to the enzalutamide treatment: C4-2 cells treated with DMSO (group A, C4-2_D + /E-_), C4-2 cells treated with enzalutamide (group B: C4-2_D-/E + _), C4-2^EnzR^ treated with the DMSO (group C: C4-2^EnzR^_D + /E-_), C4-2^EnzR^ treated with the enzalutamide (group D: C4-2^EnzR^_D-/E + _), EnzR-shLnc treated with DMSO (group E: EnzR-shLnc_D + /E-_), EmzR-shLnc treated with enzalutamide (group F: EnzR-shLnc_D-/E + _). Through the MTT experiment, we found that enzalutamide can affect C4-2 cells, but not C4-2^EnzR^ cells. Knockdown of PCBP1-AS1 in C4-2^EnzR^ cells can significantly inhibit cell growth, while simultaneous application of enzalutamide can achieve a more significant effect (Fig. 5G).

We further observed the effect of PCBP1-AS1 on enzalutamide resistance in vivo. We first subcutaneously transplanted tumors into nude mice, all nude mice were treated with enzalutamide or DMSO, and used different AAVs on the 16th day to verify our hypothesis (details in “Materials and methods”). The results of in vivo experiments showed that enzalutamide had no significant effect on drug-resistant xenograft tumors. After knocking down PCBP1-AS1 (Fig. 5J), the sensitivity of tumors to enzalutamide increased significantly (Fig. 5H, I). The tumor volume in the AAV-PCBP1-AS1 group began to decline on the 22nd day, and subsequent immunohistochemistry also confirmed that after knocking down PCBP1-AS1, tumor AR, AR-V7, and Ki-67 expressions were significantly reduced (Fig. 5K). Overall, our results show that targeting PCBP1-AS1 can significantly increase the sensitivity of prostate cancer to enzalutamide.

## Discussion

**Fig. 6:**
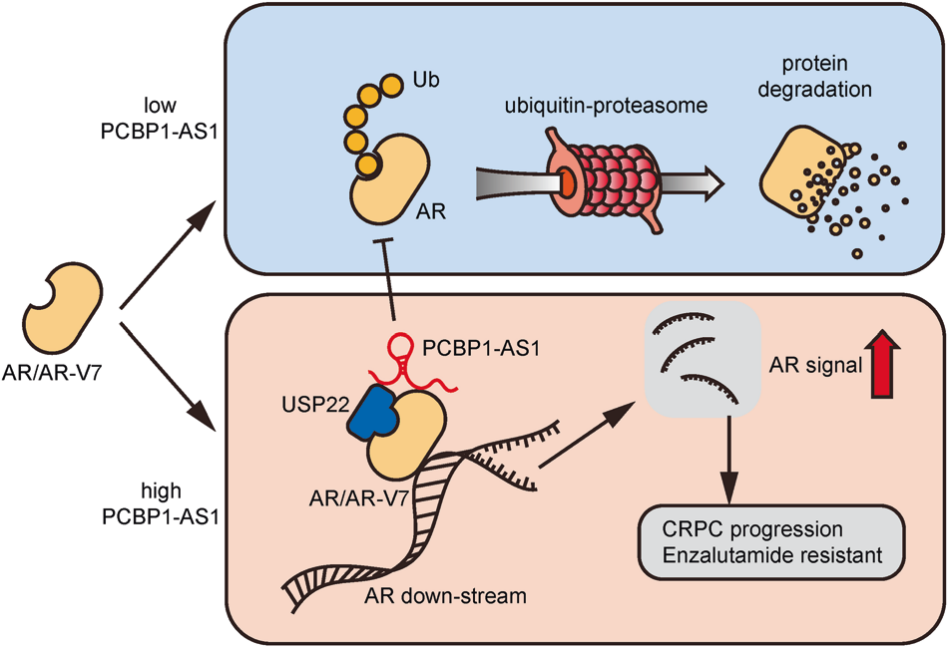
PCBP1-AS1 promotes AR/AR-V7 deubiquitination and enzalutamide resistance by enhancing USP22-AR/AR-V7 complex stability. During CRPC, upregulated PCBP1-AS1 binds to USP22 and the NTD domain of AR/AR-V7, subsequently promotes AR/AR-V7 stability, represses AR ubiquitination, and promotes CRPC enzalutamide resistance.

In this study, we identified PCBP1-AS1 as a novel regulator of AR and AR-V7 by enhancing deubiquitination through stabilizing the USP22-AR/AR-V7 complex. PCBP1-AS1 is closely related to patients’ clinical outcomes, targeting PCBP1-AS1 can inhibit tumor growth and enzalutamide resistance. Which may provide new sight in treatment against CRPC (Fig. 6).

Researches about PCBP1-AS1 mainly focused on its role in malignant disease. Most of this research identified PCBP1-AS1 as a biomarker or a factor in ceRNA networks. Till present, PCBP1-AS1 was identified to be functional or a biomarker in hepatocellular cancer, oral carcinoma, and Hodgkin lymphoma [21–23]. However, these studies are not in-depth. In this research, PCBP1-AS1 are usually identified by bioinformatics or high-throughput sequencing, and only a few phenotypic experiments are used to confirm its functions, and its mechanisms and possible clinical application values have not been discussed in-depth. However, it is worth noting that in hepatocellular carcinoma, the relevant mechanism of PCBP1-AS1 has been reported [23]. In this study, PCBP1-AS1 was reported to promote the progression of hepatocellular carcinoma by interacting with PCBP1 to regulate the PRL-3/AKT signaling pathway. In general, the studies on PCBP1-AS1 are not detailed, and there is no in-depth study on PCBP1-AS1 in prostate cancer. Therefore, our research is innovative. Through in vivo and in vitro experimental verification, database retrieval, and sequence-based computer prediction methods, we have revealed the association between PCBP1-AS1 and AR and AR-V7 for the first time, and further explored the possibility of PCBP1-AS1 in the application of medical transformation. At present, enzalutamide treatment of CRPC is facing drug resistance, and to a certain extent, our research provides new options for the treatment methods that are currently scarce [12, 24, 25].

The research of LncRNA in prostate cancer, especially CRPC has been relatively mature, and previous studies have also shown the broad prospects of lncRNA in the treatment of prostate cancer[26]. PCA3 is one of the objects studied earlier. Traditional prostate cancer generally uses PSA as a detection indicator, and with the implementation of clinical applications, the shortcomings of PSA’s lack of specificity have become increasingly significant [27]. The high specificity of PCA3 can better solve the problems faced by PSA. Many clinical studies have shown that PCA3 can be detected in urine, which makes this noninvasive detection method an alternative to PSA [14, 28, 29]. Another in-depth study of lncRNA is HOTAIR. HOTAIR prevents the degradation of AR by the ubiquitin–proteasome pathway by antagonizing the binding of MDM2 and AR and participates in promoting the transcription of AR target genes [15]. In addition, in recent years, lncRNAs such as PCAT1 and GAS5 have also been reported to participate in the regulation of the progress of CRPC through different mechanisms [16, 30]. In short, the research of lncRNA in prostate cancer has made progress, but related fields still need to be further explored. Our study further expands the function of lncRNA from the perspective of deubiquitination. By confirming that PCBP1-AS1 stabilizes the USP22-AR/AR-V7 complex, it improves the mechanism of lncRNA post-translational regulation of CRPC, which may provide new ideas for the diagnosis and targeted therapy of CRPC.

Ubiquitination is an important post-translational protein modification and is involved in the regulation of many eukaryotic signal pathways [31]. Abnormal ubiquitin signals might be the molecular causality of certain cancers, neurodegenerative diseases, or cardiovascular diseases [32]. Ubiquitination-related studies may shed light on the molecular causes of abnormal changes in tumor suppressors or oncoproteins. In general, ubiquitin is formed by covalently coupling to lysine side-chain residues through a series of enzymatic reactions, and multiple ubiquitin can be combined to the same protein [33]. The human genome contains ~50 genes encoding E2 enzymes and 600 genes encoding E3 ligases. Similarly, there are more than 90 deubiquitinating enzymes (DUB) that can inhibit ubiquitination and separate ubiquitin from protein [34]. At present, the known deubiquitinating enzymes have the most members and the most diverse structure is the ubiquitin-specific protease (USPs). Most USPs regulate tumor formation and progression by modulating substrates. USP1 function as an oncogene by interacting with DNA repair signal through PCNA and FANCD2 [35], USP4 promote cancer progression by deubiquitinating TRAF2 and TRAF6 and thereby regulating the TGFβ signaling pathway [36]. In prostate cancer, USP14, USP22, and USP26 were reported to play roles in cancer progression. USP14 has been reported to compete with MDM2 to bind to AR and prevent the ubiquitination and degradation of AR [37]. USP26 has been reported to bind to AR through LXXLL and FXXFF motifs to enhance AR deubiquitination and enhance AR downstream transcription [20]. USP22 has been reported to promote tumor progression by participating in the mediation of DNA repair processes [38]. In addition, USP22 has also been reported to be related to the ubiquitination of AR and AR-V7 in CRPC, but how this enhanced connection in CRPC was generated has not been an In-depth study [19]. The potential of this regulation for clinical applications has also not been revealed. In this study, we proved that the important factor for the stability of the USP22-AR/AR-V7 complex in CRPC is the high expression of PCBP1-AS1. Targeting PCBP1-AS1 can significantly reduce the combination of USP22 and AR or AR-V7 and reduce the stability of the USP22-AR/AR-V7 complex. The destruction of this stabilization mechanism can significantly increase the sensitivity of tumors to enzalutamide. Therefore, the combined application of enzalutamide and targeting PCBP1-AS1 may provide new ideas for the clinical treatment of refractory CRPC.

Our research also has shortcomings. First, the application of PCBP1-AS1 is still under discussion. It may be feasible to use antisense oligonucleotide (ASO) to construct molecular drugs and target them by oncolytic viruses. But at present, the biological safety of ASO drugs and oncolytic viruses is not yet fully understood, and sufficient postmortem demonstrations are still needed before conducting clinical studies. In addition, the functions of genes are diversified. This study only reveals the mechanism of PCBP1-AS1 in the AR signaling pathway, the possibility that PCBP1-AS1 can regulate CRPC in an AR-independent manner cannot be denied. Third, this study only explored AR-V7, whether PCBP1-AS1 can regulate other AR splicing variants that contain intact NTD is still unknown (such as AR-V567es). In any case, our research is a breakthrough. For the first time, we have revealed the important mechanism of PCBP1-AS1 in the AR signaling pathway and its application potential in refractory CRPC, which has high clinical application value.

## Conclusion

In this study, we first revealed the important function of long noncoding RNA PCBP1-AS1 in prostate cancer. PCBP1-AS1 stabilizes the USP22-AR/AR-V7 complex, enhances the deubiquitination of AR and AR-V7, and promotes the progress of CRPC. Targeting PCBP1-AS1 can significantly enhance the sensitivity of tumors to enzalutamide in vivo and in vitro and can provide new possibilities for clinical diagnosis and targeted therapy of refractory CRPC.

## Materials and methods

### Data collection and tissue microarray

TCGA dataset was downloaded from the UCSC-XENA website. GSE124291 dataset was downloaded from the GEO website, which contains three repeats of LNCaP-AI cells and three repeats of LNCaP cells. As for tissue microarray, 4 BPH patients, 28 HSPC patients, and 12 CRPC patients were collected from the Affiliated Cancer Hospital of Zhengzhou University. All patients are informed and agree to our request.

### Primers, reagents, antibodies, and plasmids

All detailed information can be found in Supplementary Table 1.

### Cell culture, LNCaP-AI and C4-2^EnzR^ formation

LNCaP and C4-2 cells were donated by Zhang Mingpeng, Institute of Urology, Tianjin Medical University Second Hospital. LNCaP-AI cells were generated from LNCaP cells. We use 1640 medium containing 10% carbon-adsorbed fetal bovine serum to continuously culture LNCaP cells to stable passages for 50 generations. When the cells can grow normally in this environment, we named this cell LNCaP-AI cell, and conduct follow-up experiments.

C4-2^EnzR^ cells are induced from C4-2 cells. We first added 1 µM of enzalutamide to the normally cultured C4-2 cells. After the remaining cells were able to grow normally, we gradually increased the concentration of enzalutamide to 2 µM, 5 µM, 10 µM, 20 µM. Subsequently, we continued to culture the cells in the environment of 20 μM enzalutamide to stable passage for 50 generations. At this time, we believed that C4-2 cells had become resistant to enzalutamide and named C4-2^EnzR^.

As for cell culture, all cells were cultured in 37 °C cell incubator under 5% CO_2_. 1640 with 10% fetal bovine serum was used for C4-2 and LNCaP cells, 1640 with 10% carbon-absorbed fetal bovine serum was used for LNCaP-AI cells.

### Protein extraction and western blot

The extraction of cell and tissue proteins is done using RIPA. For the cells, the culture medium was first discarded and washed with PBS, then an appropriate amount of RIPA was added, placed at 4 °C for 30 min shaking, and centrifuged at 12,000 rpm for 30 min at 4 °C. Subsequently, the supernatant was aspirated, the protein concentration was determined by the Bradford method, and stored at −80 °C for later use.

For western blot, mix the protein and 5× loading buffer at a ratio of 4:1 and heat at 95 for 5 min. The samples were then added to a 10% SDS-PAGE gel for electrophoresis. Use PVDF membrane for electroporation, keep it at a constant current of 250 mA for 120 min, then block with 5% skimmed milk powder for 1 h, rinse with TBST three times, and incubate overnight with the corresponding primary antibody at 4 °C. On the second day, remove the primary antibody, rinse with TBST three times, then incubate with the corresponding secondary antibody for 1 h, then rinse with TBST three times before exposure.

### RNA extraction and qPCR

After collecting the cells, add the appropriate amount of Trizol reagent, mix well, add 200 µL of chloroform, mix well and place at 4 °C for 15 min, then centrifuge at 12,000 rpm at 4 °C for 15 min, collect 500 µL of supernatant and add the same amount of isopropyl alcohol, mix well and stand at 4 °C for 10 min, then centrifuge at 12,000 rpm at 4 °C for 10 min, discard the supernatant, add 1 mL of 70% absolute ethanol, after shaking, centrifuge at 7500 rpm at 4 °C for 5 min, discard the supernatant and dry, Then add 20 μL of enzyme-free water, measure the concentration and place it at −80 °C for subsequent use.

cDNA reverse transcription uses Thermo RevertAid First Strand cDNA Synthesis Kit, and all steps are performed strictly in accordance with the corresponding instructions. For qPCR, mix according to the following recipe: 10 µL FastStart Universal SYBR Green Master (Rox), 2 µL cDNA, 1 µL preprimer, 1 µL post primer, 6 µL enzyme-free water. After mixing uniformly, add it to a 96-well plate for qPCR, and then use the corresponding machine for qPCR detection.

### RNA immunoprecipitation (RIP) and co-immunoprecipitation (co-IP)

RIP assay was performed using Magna RIP kit, all detail steps were done according to the corresponding manuscripts. After RNA was extracted from beads, the following step should be cDNA synthesis and PCR or qPCR.

Co-IP assay was performed using Thermo Pierce Classic Magnetic IP/Co-IP Kit. all detail steps were done according to the corresponding manuscripts. After protein was extracted from beads, western blot was used as the detection method.

It is important to point out that all the antibodies used here should be suitable for RIP or co-IP, otherwise it may lead to failure to obtain correct results.

### RNA pulldown

RNA pull-down experiment consists of five parts, plasmid digestion, in vitro transcription, 3’-end biotinylation labeling, RNA pulldown, and western blot detection. The plasmid containing PCBP1-AS1 sequence was generated and digested by Sangon Biotech (Sangon Biotech (Shanghai)Co., Ltd), T7 promoter was added to facilitate our subsequent in vitro transcription. In vitro transcription was performed with MEGAscript™ T7 Transcription Kit, 3’ end biotinylation was done by Pierce RNA 3’ End Biotinylation Kit, RNA pull-down assay was performed by Pierce Magnetic RNA-Protein Pull-Down Kit. All steps above followed strictly to the corresponding manuscript. After protein was extracted, western blot was followed as a detection method.

### In vivo experiment

This study contains two parts of animal experiments, using C4-2 cells and C4-2^EnzR^ cells, respectively. The two parts of the experiment have different purposes. The C4-2 cell-related animal experiment aims to observe the effect of targeting PCBP1-AS1 on tumor growth, while the C4-2^EnzR^-related animal experiments focused on the effect of targeting PCBP1-AS1 on the sensitivity of tumor enzalutamide.

As for tumor growth, after collecting the cells, they were inoculated subcutaneously in ten 8-week-old nude mice, each inoculated with 1 × 10^7^ cells. After a week, tumors were formed. The tumor size was measured every other day, and adeno-associated virus (AAV) was injected into the tumor on the 14th day—five injected with AAV targeting PCBP1-AS1 and five with AAV containing invalid sequences—continue to cultivate the mice, and count the tumor volume. After 1 week, the mice were euthanized under anesthesia, and then the tumors were taken out and photographed and immersed in formalin for short-term preservation, and then made into wax blocks for subsequent experiments (Supplementary Fig. 1A).

As for enzalutamide-related in vivo experiments, C4-2^EnzR^ cells were collected and inoculated subcutaneously in ten 8-week-old nude mice, each inoculated with 1 × 10^7^ cells. From the 7th day, enzalutamide was administered once a day via oral gavage at 10 mg/kg in 1% carboxymethyl cellulose, 0.1% Tween-80, and 5% DMSO. One week later, the ten nude mice were divided into two groups (five each group) and injected with AAV-PCBP1-AS1 or AAV-Scramble, keep feeding under enzalutamide condition for 2 weeks, mice were euthanized by the same method, Take out the tumor and take pictures, and save the tumor in formalin for a short time for the subsequent experiments (Supplementary Fig. 1B).

### Statistics and code availability

The bioinformatics analysis in this study was performed using R-Version 4.0.0, and the R packages used were limma, edgeR, ggplot2, survminer, and Survival. In terms of statistical analysis, mean + /− SD is used, the significance between the two groups is performed by *T* test, and the significance between multiple groups is calculated by ANOVA. The significance of Kaplan–Meier survival analysis was calculated using the log-rank test. All codes can be traced, please contact zhangboya_0722@163.com.

### Experimental replicates and *P* value significance

For the TMA experiments in Fig. 1A–H and Fig. 3A, B, each tissue include 2 dots on the array, used as experimental replicates. For the in vitro cell experiments, including Fig. 2A–G, Fig. 3C–L, Fig. 4A–I, and Fig. 5A–G, all experiments were repeated three times. For in vivo experiments, including Fig. 2H–K and Fig. 5H–K, all experiments have at least five biological replicates and two experimental replicates.

Significance: *P<0.05, **P<0.01, ***P<0.001, ****P<0.0001.

## Supplementary information

Reproducability Checklist

Supplementary figure 1

Supplementary table 1

## Data Availability

All raw data and code are available. Please contact zhangboya_0722@163.com or zlyyyaoweitao1402@zzu.edu.cn for raw data and code.
