# Supplementary material for: LncRNA PCBP1-AS1-mediated AR/AR-V7 deubiquitination enhances prostate cancer enzalutamide resistance
Source: Cell Death Dis. 2021 Sep 20;12(10):856. doi: 10.1038/s41419-021-04144-2 (PMC8452729; doi:10.1038/s41419-021-04144-2)
Supplement: Supplementary file 2 — Supplementary figure 1 [file 41419_2021_4144_MOESM2_ESM.pdf]

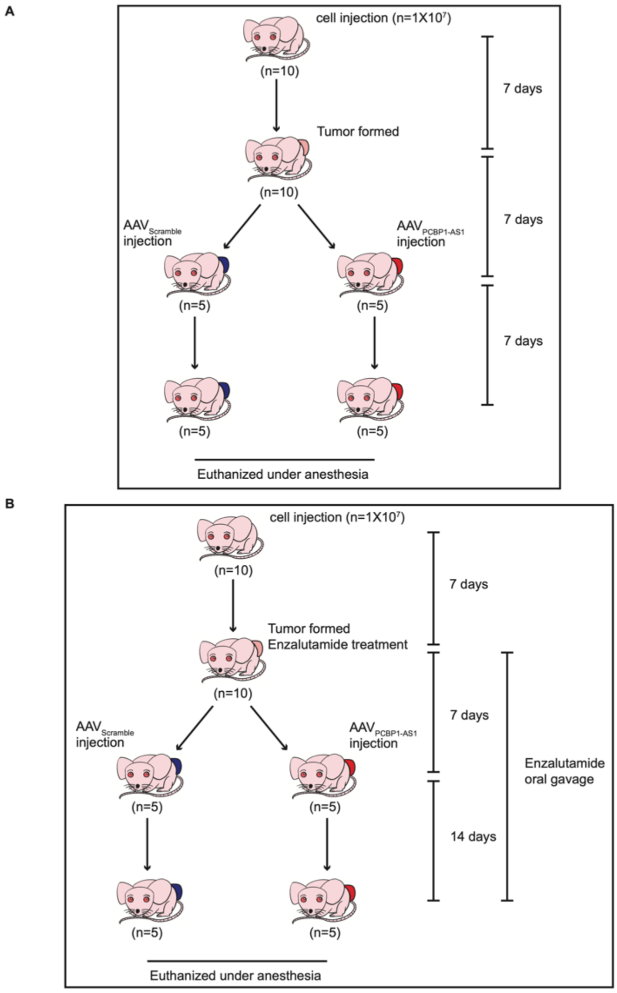

Supplementary figure 1: *in vivo* experiments workflow. A: Workflow of tumor growth in vivo experiment. Number of nude mice used were 10. In general, after collecting the cells, they were inoculated subcutaneously in ten 8-week-old nude mice. After a week, tumors were formed. The tumor size was measured every other day, and adeno-associated virus (AAV) was injected into the tumor on the 14th day. five injected with AAV targeting PCBP1-AS1 and five with AAV containing invalid sequences. continue to cultivate the mice and count the tumor volume. After 1 week, the mice were euthanized under anesthesia. Details can also be found in materials and methods. **B**: As for enzalutamide related in vivo experiments, C4-2^{EnzR} cells were collected and inoculated subcutaneously in ten 8-week-old nude mice, each inoculated with $1X10^7$ cells. From the 7th day, Enzalutamide was administered once a day via oral gavage at 10mg/kg in 1% carboxymethyl cellulose, 0.1% Tween-80, and 5% DMSO. One week later, the ten nude mice were divided into two groups (five each group), and injected with AAV-PCBP1-AS1 or AAV-Scramble, keep feeding under enzalutamide condition for two weeks, mice were euthanized by the same method. Details can also be found in materials and methods.
