# Supplementary material for: LncRNA PCBP1-AS1-mediated AR/AR-V7 deubiquitination enhances prostate cancer enzalutamide resistance
Source: Cell Death Dis. 2021 Sep 20;12(10):856. doi: 10.1038/s41419-021-04144-2 (PMC8452729; doi:10.1038/s41419-021-04144-2)
Supplement: Supplementary file 3 — Supplementary table 1 [file 41419_2021_4144_MOESM3_ESM.docx]

**Supplementary table.1: Primers, reagents, antibodies and plasmids**

**Primers**

| Gene | Subtypes | Sequence |
| --- | --- | --- |
| PCBP1-AS1 | Forward | ACTACTCAGTCAATTGCTCCA |
| PCBP1-AS1 | Reverse | ATTTCCTTACTGACCTGCAT |
| AR | Forward | CTACATCAAGGAACTCGATCGT |
| AR | Reverse | CATGTGTGACTTGATTAGCAGG |
| KLK3 | Forward | GCCTGGATCTGAGAGAGATATCATC |
| KLK3 | Reverse | ACACCTTTTTTTTTCTGGATTGTTG |
| FKBP5 | Forward | GGTTCCTGGGCAGGAGTAAG |
| FKBP5 | Reverse | AACGTGGATCCCACACTCTC |
| GAPDH | Forward | AGCCACATCGCTCAGACAC |
| GAPDH | Reverse | GCCCAATACGACCAAATCC |

**shRNAs and AAVs**

| Gene | Sequence |
| --- | --- |
| PCBP1-AS1 | GGATCCGCCACAGCAGCACAGAATTCTTTCAAGAGAAGAATTCTGTGCTGCTGTGGCTTTTTTA |

**Antibodies**

| Antibody | Corporation | Cat No | Application | Dilution ratio/Dosage |
| --- | --- | --- | --- | --- |
| AR | Abcam | Ab108341 | WB/IHC | 1:5000 |
| AR (IP specific) | Abcam | Ab226171 | RIP/co-IP | 5μg |
| AR-V7 | Abcam | Ab198394 | WB/IHC | 1:500 |
| FKBP5 | Abcam | Ab126715 | WB | 1:5000 |
| KLK3 | RevMAb | 31-1210-00 | WB | 1:1000 |
| Ubiquitin | Abcam | Ab134953 | WB | 1:5000 |
| MYC-Tag | CST | 2276S | RIP-qPCR  RNA pulldown | 5μg |
| USP22 | Abcam | Ab109435 | WB | 1:2000 |
| USP22(IP specific) | Abcam | Ab195289 | RIP/co-IP | 5μg |
| USP5 | Abcam | Ab154170 | WB | 1:5000 |
| OTUB1 | Abcam | Ab175200 | WB | 1:1000 |
| MDM2 | Abcam | Ab16895 | WB | 1:1000 |
| GAPDH | Abcam | Ab181602 | WB | 1:10000 |
| IgG | Abcam | Ab172730 | RIP/co-IP | 5µg |
| Ki-67 | Abcam | Ab15580 | IHC | 1:200 |

**Reagents:**

| Reagent name | Corporation | Cat No |
| --- | --- | --- |
| RNAscope Multiplex Fluorescent Reagent Kit v2 | Advanced Cell Diagnostics | 323100 |
| TRIzol Reagent | Invitrogen | 15596026 |
| Thermo RevertAid First Strand cDNA Synthesis Kit | Thermo Scientific | K1621 |
| FastStart Universal SYBR Green Master (ROX) | Roche | 4913914001 |
| Methylthiazolyldiphenyl-tetrazolium bromide (MTT) | Merck | 57360-69-7 |
| Crystal Violet | Sigma-Aldrich | 549-62-9 |
| Immunohistochemistry detection system | ZSGB-Bio | PV-6000 |
| Hematoxylin-eosin staining reagent | Solarbio Life Science | G1121 |
| Magna RIP™ RNA-Binding Protein Immunoprecipitation Kit | Merck | 17-700 |
| MEGAscript™ T7 Transcription Kit | Invitrogen | AM1334 |
| Pierce™ RNA 3' End Biotinylation Kit | Thermo Scientific | 20160 |
| Pierce™ Magnetic RNA-Protein Pull-Down Kit | Thermo Scientific | 20164 |
| MG-132 | Selleckchem | S2619 |
| Chloroquine | Sigma-Aldrich | C6628 |
| CHX | Sigma-Aldrich | C7698 |
| SDS-PAGE Gel Kit | Solarbio Life Science | P1200 |
| Pierce™ Classic Magnetic IP/Co-IP Kit | Thermo Scientific | 88804 |
